# Islet-expressed circular RNAs are associated with type 2 diabetes status in human primary islets and in peripheral blood

**DOI:** 10.1186/s12920-020-0713-2

**Published:** 2020-04-20

**Authors:** Shahnaz Haque, Ryan M. Ames, Karen Moore, Benjamin P. Lee, Nicola Jeffery, Lorna W. Harries

**Affiliations:** 1 RNA-Mediated Mechanisms of Disease Group, Institute of Biomedical and Clinical Sciences, University of Exeter Medical School, University of Exeter, RILD South, Barrack Road, Exeter, EX2 5DW UK; 2 Biosciences, University of Exeter, Exeter, UK; 3 College of Life and Environmental Sciences, University of Exeter, Exeter, UK

**Keywords:** circRNA, Diabetes, Human, islet, EndoC-βH1 beta cells

## Abstract

**Background:**

Circular RNAs are non-coding RNA molecules with gene regulatory potential that have been associated with several human diseases. They are stable and present in the circulation, making them excellent candidates for biomarkers of disease. Despite their promise as biomarkers or future therapeutic targets, information on their expression and functionality in human pancreatic islets is a relatively unexplored subject.

**Methods:**

Here we aimed to produce an enriched circRNAome profile for human pancreatic islets by CircleSeq, and to explore the relationship between circRNA expression, diabetes status, genotype at T2D risk loci and measures of glycaemia (insulin secretory index; SI and HbA1c) in human islet preparations from healthy control donors and donors with type 2 diabetes using ANOVA or linear regression as appropriate. We also assessed the effect of elevated glucose, cytokine and lipid and hypoxia on circRNA expression in the human beta cell line EndoC-βH1.

**Results:**

We identified over 2600 circRNAs present in human islets. Of the five most abundant circRNAs in human islets, four (*circCIRBP*, *circZKSCAN*, *circRPH3AL* and *circCAMSAP1*) demonstrated marked associations with diabetes status. C*ircCIRBP* demonstrated an association with insulin secretory index in isolated human islets and *circCIRBP* and *circRPH3AL* displayed altered expression with elevated fatty acid in treated EndoC-βH1 cells. *CircCAMSAP1* was also noted to be associated with T2D status in human peripheral blood. No associations between circRNA expression and genotype at T2D risk loci were identified in our samples.

**Conclusions:**

Our data suggest that circRNAs are abundantly expressed in human islets, and that some are differentially regulated in the islets of donors with type 2 diabetes. Some islet circRNAs are also expressed in peripheral blood and the expression of one, *circCAMSAP1,* correlates with diabetes status. These findings highlight the potential of circRNAs as biomarkers for T2D.

## Background

One of the key difficulties in dissecting the factors driving progression of multifactorial polygenic chronic diseases such as type 2 diabetes (T2D) is the degree of heterogeneity that it presents. Although the development of diabetes like other common chronic disorders has a large lifestyle contribution, there is a substantial genetic component [1]. 70% of individuals with prediabetes eventually develop diabetes [2, 3], with increasing evidence suggesting that diabetic complications such as peripheral nephropathy and retinopathy may initiate at the pre-diabetic stage [2]**.** Identifying people at risk of type 2 diabetes, or those likely to progress from impaired glucose tolerance to overt disease is thus an important aim. Understanding the molecular causes of T2D, and identification of sensitive and specific biomarkers to indicate those at risk of pre-diabetes, or of transition from pre-diabetes to overt disease is therefore a key aim for research.

Genome wide association studies (GWAS) for T2D have identified over 143 risk loci associated with susceptibility to T2D [1]. More than 85% of these disease-associated variants reside in non-coding regions of the genome [4]. Over 80% of the human genome is predicted to display some degree of functionality [5], so it is likely that many of the diabetes-associated genetic variants may act via dysregulation of gene expression. Disruption of the activity or function of non-coding RNAs that moderate gene activity, such as microRNA (miRNA) or long non-coding RNA (lncRNA) may have particular relevance [6].

Circular RNAs (circRNAs) are an emerging class of non-coding RNA (ncRNA) generated by the back splicing of downstream exons to the 3′ acceptor splice site of upstream exons and result in a covalently closed circular structure containing one or more exons [7]. Their mode(s) of action remain to be fully elucidated but they have been suggested to manipulate gene expression by moderation of transcription [8], interaction with cellular proteins [9], sequestration of RNA-binding proteins [10] or sponging miRNA [11]. Their covalently closed structure means that they are resistant to exonucleases; accordingly, they have half-lives on average 19–24 h [12], being significantly more stable than linear mRNAs from their cognate genes which have half-lives typically in the region of 4–9 h [13]. Data on circRNA abundance can be extracted from conventional NGS data, but such data may also include aberrant back spliced sequences from linear transcripts as well as genuine circRNAs.

CircRNAs may have potential as biomarkers for the development of diabetes or as future molecular targets for novel diabetes therapeutics [14–16]. An important pre-requisite for this is the characterisation of circRNA sequences in diabetes-relevant tissues such as pancreatic islets and in more accessible tissues such as peripheral blood. Cell-type specific circRNA expression has previously been reported in human pancreatic α, β and δ cells [17], but these profiles were not circRNA-specific, being extracted from published NGS data in the absence of RNase R treatment to remove linear RNA. Other human islet circRNA profiles have been generated using microarray approaches, which will capture only known circRNAs [18].

We present here an enriched whole circRNAome profile from primary human pancreatic islets which we have generated using a modified circleSeq technique [19]. This included an RNase R step to remove linear RNA and enrich for circRNAs. We firstly aimed to determine whether expression of the most abundantly-expressed islet circRNAs were associated with insulin secretory index (SI), donor HbA1c or donor diabetes status in human primary islets. Secondly, we aimed to determine whether circRNAs localising to the genomic regions encompassing the GWAS association signals for type 2 diabetes were differentially-expressed according to donor risk genotype. Thirdly, we aimed to explore whether abundantly-expressed circRNAs were responsive to diabetomimetic stimuli (hypo- or hyperglycaemia, hypoxia, elevated fatty acids or inflammatory cytokines), in the human beta cell line EndoC-βH1. Finally, we aimed to determine whether abundant islet circRNAs were differentially expressed in the peripheral blood of individuals with pre-diabetes or overt disease.

We identified 2619 circRNAs that were expressed in islet donors, 47 of which had not been previously identified in data from human tissues. 4/5 of the most abundant circRNA demonstrated differential expression in the islets of donors with T2D, whilst 2/5 demonstrated dysregulated expression in response to elements of the diabetic microenvironment in the human beta cell line EndoC-βH1 in culture. No circRNA co-localising to the GWAS signals for T2D demonstrated associations with risk genotype. Finally, 3/5 of the most abundant islet circRNAs were also expressed in blood, and the expression of one, *circCAMSAP1,* demonstrated an association with T2D status in the peripheral blood of patients with T2D, but not with impaired glucose tolerance (IGT). To conclude, we have produced the first global circRNA-only profile in human pancreatic islets, provided evidence that some are differentially expressed in the islets of donors with diabetes. One islet circRNA (*circCAMSAP1*) is also differentially expressed in the peripheral blood of subjects with T2D, highlighting a potential use for circRNA species as biomarkers of disease.

## Methods

### Pancreatic islet preparations

Snap-frozen islet preparations were purchased from ProCell Biotech (Newport Beach, CA, USA) or from the NIA IIDP resource (collected with ethical permission at source). Islet purity and viability was determined by dithizone and fluorescein diacetate/propidium iodide staining. Samples were shipped in RNAlater-ICE (Life Technologies, Carlsbad, CA, USA) to maintain transcript stability, and RNA was extracted using the total RNA extraction protocol of the miRVana miRNA isolation kit, as per the manufacturers’ instructions. Sample RNA Integrity Number (RIN) was determined using an Agilent Bioanalyser (Agilent, Santa Clara, USA). Fifty three islet samples were available from healthy donors, and 20 from donors with T2D. Islet donor characteristics are given in Table 1, with expanded information on each donor in Supplementary Table S1.

**Table 1:** Sample and donor characteristics for human islet samples used in this work

**A.**					
	**Control (*n*** **= 50)**		**T2D (*n*** **= 20)**		***p*-value**
	**Mean**	**SD**	**Mean**	**SD**	
**Age**	40.66	14.25	53.55	9.22	< 0.001
**BMI**	28.30	6.83	33.05	10.31	0.027
**SI**	2.45	1.26	–	–	–
**HbA1c**	5.44	0.36	–	–	–
**Purity**	89.28	6.00	80.38	14.92	< 0.001
**Viability**	93.53	4.80	89.73	4.54	0.003
**Sex**	F (40%);	M (60%)	F (45%)	M (55%)	0.706
**Ethnicity**	white (74%)	other (26%)	white (45%)	other (55%)	0.348
**B.**					
	**Major allele homozygotes**		**Heterozygotes and minor allele homozygotes**		***p*-value**
	**Mean**	**SD**	**Mean**	**SD**	
**rs6819243 (*CTBP1*)**					
**Age**	40.19	14.12	42.38	11.78	0.620
**BMI**	27.85	5.34	27.16	7.03	0.715
**Purity**	89.26	5.86	90.00	3.54	0.671
**Viability**	93.33	5.68	94.12	2.38	0.633
**Sex**	F (53%)	M (47%)	F (38%)	M (62%)	0.595
**Ethnicity**	white (83%)	other (17%)	white (31%)	other (69%)	0.020
**rs10758593 (*GLIS3*)**					
**Age**	41.08	15.04	40.88	12.25	0.966
**BMI**	26.27	4.14	29.07	5.80	0.130
**Purity**	89.46	4.96	88.50	6.26	0.633
**Viability**	94.81	3.09	92.33	5.37	0.133
**Sex**	F (39%)	M (62%)	F (42%)	M (58%)	0.269
**Ethnicity**	white (69%)	other (31%)	white (73%)	other (27%)	0.916
**rs7177055 (*HMG20A*)**					
**Age**	39.97	13.95	43.69	12.35	0.371
**BMI**	27.83	5.63	29.87	9.46	0.355
**Purity**	89.13	5.18	89.13	6.18	0.998
**Viability**	93.37	4.03	93.34	6.70	0.986
**Sex**	F (47%)	M (53%)	F (38%)	M (63%)	0.547
**Ethnicity**	white (72%)	other (28%)	white (75%)	other (25%)	0.697
**rs1111875 (*IDE*)**					
**Age**	48.58	9.95	33.43	12.20	< 0.001
**BMI**	30.05	8.52	26.50	4.80	0.088
**Purity**	88.61	4.91	90.57	4.73	0.175
**Viability**	94.57	3.30	93.57	4.20	0.374
**Sex**	F (58%)	M (42%)	F (30%)	M (70%)	0.056
**Ethnicity**	white (67%)	other (33%)	white (74%)	other (26%)	0.733
**rs12427353 (*SPPL3*)**					
**Age**	39.68	14.15	46.80	9.02	0.137
**BMI**	29.03	7.32	25.03	4.00	0.104
**Purity**	89.98	5.22	87.78	5.65	0.267
**Viability**	93.48	5.28	94.33	3.50	0.645
**Sex**	F (40%)	M (60%)	F (70%)	M (30%)	0.092
**Ethnicity**	white (68%)	other (33%)	white (80%)	other (20%)	0.949
**rs10203174 (*THADA*)**					
**Age**	42.18	13.62	43.36	13.38	0.802
**BMI**	29.07	7.54	25.54	5.13	0.155
**Purity**	89.64	4.50	89.09	6.25	0.754
**Viability**	93.85	4.25	94.64	2.46	0.565
**Sex**	F (47%)	M (53%)	F (36%)	M (64%)	0.546
**Ethnicity**	white (74%)	other (26%)	white (55%)	other (45%)	0.920

A. Characteristics of human islet preparations from donors with (*n* = 20) and without (*n* = 50) T2D. B. Characteristics of human islet preparations from control donors used for GWAS genotype analysis (*n* = 53). SD = standard deviation. Islet sets for parts A and B are only partly overlapping; non-diabetic samples used for assessment of effects of diabetes status were selected from the larger pool to allow for matching of islet donors with and without diabetes. Differences in parameters between islet groups was determined by t-test. B. Characteristics of human peripheral blood donors with (*n* = 20) and without (*n* = 50) T2D

### Generation of human primary islet circRNA profile

For our initial description of the islet circRNome, we generated circular RNA profiles from a pooled sample consisting of 5 individual islet preparations from donors without T2D using a modified ‘CircleSeq’ procedure [20] as in our previous work [21]. Samples were derived from 3 females and 2 males, with an average age of 50.2 years and an average BMI of 26.4, and were pooled to prevent any bias arising from signals arising from single donors. Islet preparations had, an average viability 94.4% and an average purity of 81%. CircRNA sequencing was carried out as previously published [21]. Sixty-two M reads were obtained from the RNase R-treated sample and 41 M reads from the mock-treated sample. The mean Q score was 38.9–39.1 and the total error rate was 0.24%. CircRNA sequences were then determined and quantified as in our previous work [21].

### Pathway analysis of genes hosting differentially-regulated circRNA

To determine whether circRNAs identified were derived from genes clustered into specific gene ontology pathways, we carried out an initial gene set enrichment GSE analysis using the ClueGO Cytoscape version 2.5.2 plug-in (Bindea et al., 2009), as described in our previous work [21].

### Selection of circRNA for validation

CircRNA were selected for follow up on two criteria. Firstly, levels of individual circRNA in the islet were ranked by abundance. We selected the 5 circRNAs most abundantly expressed in islets (*circCAMSAP1, circCIRBP, circRHOBTB3, circRPH3AL and circZKSCAN1*) for further analysis. We also assessed the expression of the linear reference transcript for each circRNA in each case. The second class of circRNAs selected for follow up were those mapping to the GWAS loci for T2D.

The circRNA profile in our study was mapped against the T2D susceptibility loci [22]. The co-ordinates of the upstream and downstream exons predicted to constitute each circRNA were then cross-referenced against the T2D GWAS signals using a custom Python 2.7 script to determine whether any circRNAs co-localized within the recombination windows. Thirteen circRNAs fulfilled these criteria and were selected for follow up (*circCTBP1_1, circCTBP1_2, circGLIS3, circHMG20A, circIDE1, circIDE2, circSPPL3_1, circSPPL3_2, circTHADA1, circTHADA2, circTHADA3, circTHADA4 and circTHADA5*). The expression of both circRNA and their host linear transcripts were assessed.

### Design of RT-qPCR assays for circRNA validation

We designed and validated custom quantitative RT-qPCR assays specific to the unique back-spliced junctions of each circRNA to be followed up. Assay sequences are given in Supplementary Table S2. Probes and primers were placed to avoid genetic variation. Assay efficiency, linear range and accuracy were determined by 1:10 serial dilutions of synthetics oligonucleotides corresponding to each back spliced junction (ThermoFisher, Foster City, USA).

### Reverse transcription

cDNA synthesis for analysis of circRNA expression in islets, EndoC-βH1 cells and across a panel of tissues was carried out using the Superscript® VILO™ cDNA synthesis kit (ThermoFisher, Foster City, USA) according to manufacturer’s instructions. Reactions contained 100 ng/μL RNA in a final reaction volume of 20 μL. Reaction conditions were 25 °C for 10 min, 42 °C for 60 min and 85 °C for 5 min.

### Expression of islet circRNAs in other tissues

The expression of the 13 circRNAs co-localizing to T2D-GWAS loci and the 5 most abundant circRNAs expressed in pancreatic islets and their parent linear transcripts were assessed in 39 different tissues by quantitative RT-qPCR. The tissue panel was commercially-sourced panel consisting of pooled samples from different donors (tissue RNA samples were sourced from Ambion (Bath, UK), Biochain (Newark, USA) or BD Biosciences (Swindon, UK). Reaction mixes contained 2.5 μL Taqman® Universal PCR mastermix II, no AmpErase® UNG, (ThermoFisher, Foster City, USA), 1.75 μL dH_2_O, 0.5 μL cDNA and 0.25 μL Taqman® gene expression assay (ThermoFisher, Foster City, USA) in a 5 μL final reaction volume. Cycling conditions were 50 °C for 2 min, 95 °C for 10 min and 50 cycles of 15 s at 95 °C for 30 s and 1 min at 60 °C. Reactions were carried out on the 12 K Flex platform (ThermoFisher, Foster City, USA) in 3 technical replicates. Target abundance was assessed using the Comparative Ct method, and was expressed relative to the geometric mean of the target and control set as a whole, since endogenous controls alone did not provide a robust baseline. Data for each target within the tissue panel was then normalised to its median level of expression across the entire panel.

### Assessment of associations between the islet expression of abundant circRNAs, insulin secretory index (SI), HbA1c or T2D status

RNA samples and clinical data were available for islet preparation from 50 non-diabetic donors and from 20 donors with T2D. Islet donor characteristics are given in Table 1. We assessed the expression of the 5 most abundant circRNAs expressed in pancreatic islets as well as their host linear transcripts in relation to insulin secretory index, HbA1c or diabetes status in these samples by quantitative RT-qPCR. Reaction mix contained 2.5 μL Taqman® Universal PCR mastermix II, no AmpErase® UNG, (ThermoFisher, Foster City, USA), 1.75 μL ddH_2_O, 0.5 μL cDNA and 0.25 μL Taqman® gene expression assay (ThermoFisher, Foster City, USA) in a 5 μL final reaction volume. Cycling conditions were 50 °C for 2 min, 95 °C for 10 min and 50 cycles of 15 s at 95 °C for 30 s and 1 min at 60 °C. Reactions were carried out on the 12 K Flex platform (ThermoFisher, Foster City, USA) in 3 technical replicates. Target abundance was assessed using the Comparative Ct method, and expressed relative to the geometric mean of the assay set. Levels of target expression in the islets of donors with T2D were then normalised to the median level of that transcript in non-diabetic islets controls. For assessment of SI or HbA1c, expression was normalised to the median level of each circRNA in control samples. Differential expression by diabetic status, SI or HbA1C was then assessed by one way ANOVA using StataSE15 (StataCorp, Texas, USA), with adjustment made for potential confounders including age, sex, BMI and ethnicity.

### Determination of donor genotype at T2D risk SNPs

RNA samples and phenotypic data were available from 53 non-diabetic islet donors. Characteristics of participants are given in Table 1 and supplementary Table S1. The expression of 13 circRNAs co-localising to the genomic regions containing the GWAS association loci for T2D was assessed in relation to genotype. Genotype at the GWAS association loci for T2D with expression of circRNAs located in those regions was accessed by virtue of the small amounts of genomic DNA which are co-eluted in RNA preparations upon RNA extraction. We used a whole genome amplification (WGA) approach to amplify co-eluted DNA for genotyping using the REPLI-g Mini kit (Qiagen, Paisley, UK). WGA was carried out using 2.5 μL RNA and was performed according to manufacturer’s instructions. Genotype was then determined by Sanger Sequencing of PCR amplicons containing the SNP in question. PCR reaction mixes included 2.4 μL primer mix containing a 1:1 ratio of forward: reverse primers (ThermoFisher, Foster City, USA), 4 μL MegaMix-Royal (Microzone ͧ, Brighton, UK) and 1.60 μL cDNA in a final reaction volume of 8 μL. Reaction condition for PCR were 95 °C for 12 min, 40 cycles for 95 °C for 30s, annealing for 1 min, 72 °C for 1 min followed by 72 °C for 10 min**.** In one case, sequence analysis proved inconclusive. In this case, genotype was determined by qPCR with TaqMan® Genotyping assay. Reactions contained 2.5 μL TaqMan® Genotyping Master Mix (ThermoFisher, Waltham, MA, USA), 0.25 μL Taqman® genotyping assay (rs6819243) (ThermoFisher, Waltham, MA, USA), 1.75 μL dH_2_O and 0.5 μL whole genome amplified template in a 5 μL final reaction volume. Target abundance was assessed using the Comparative Ct method, and expressed relative to the geometric mean of the target and control set as a whole. The expression of each target was then normalised to median levels of that target across the collection. Expression levels were then related to genotype of the islet donors by one way ANOVA using StataSE15 (StataCorp, Texas, USA) with adjustment for age, sex, BMI and ethnicity.

### Assessment of circRNA expression in EndoC-βH1 under diabetomimetic conditions

The expression levels of the 5 circRNAs chosen on the basis of islet abundance and their linear transcripts were also assessed in the human pancreatic beta cell line EndoC-βH1, following exposure to dysregulated glucose (2.5 mM and 25 mM), hypoxia (1% O_2)_, dyslipidaemia (0.5 mM palmitic acid) or proinflammatory cytokines (TNFα (1000 U/ mL, INFγ (750 U/ mL) and IL1β (75 U/ mL) as described in our previous work [23]. Analysis was from RNA from different time points selected to exclude effects due to compromised cell viability (up to 48 h for glycemia and lipid treatment, up to 36 h for cytokine treatment and up to 24 h for hypoxia exposure). CircRNA expression was measured using RT-qPCR as described above on the 12 K Flex platform (ThermoFisher, Foster City, USA)*.* Target abundance was assessed using the Comparative Ct method, and expressed relative to the geometric mean of the target and control set as a whole, since endogenous controls alone did not provide a robust baseline. Levels of each target were then normalised to the median level of each circRNA in untreated cells. Samples were run in 3 biological replicates and 3 technical replicates. Differential circRNA expression in treated cells was then assessed by one way ANOVA using StataSE15 (StataCorp, Texas, USA).

### RNA extraction from peripheral blood samples from control donors, donors with IGT and those with T2D

We assessed the expression of the 5 most abundant islet circRNAs in relation to diabetes status in RNA extracted from 285 peripheral blood samples from the Exeter 10,000 study (http://www.peninsulacrf.org/node/155). Our sample set consisted of 133 non-diabetic patients (fasting glucose < 100.8 mg/dL), 46 individuals with impaired glucose tolerance (fasting glucose 100.8 to 122.4 mg/dL) and 106 patients with overt diabetes (fasting glucose > 122.4 mg/dL). Participant characteristics are given in Table 2. This collection is a cross sectional population study consisting of samples collected from volunteer individuals living in the South West of England and recruited since 2010. Whole blood samples were collected in 2011/2012 using the PAXgene system [24] and extracted using the PAXgene Blood RNA kit (Qiagen, Paisley, UK). Written informed consent was obtained for all participants and ethical permission was granted through the National Institute for Health Research (NIHR) Clinical Facility (REC 09/H0106/75).

**Table 2:** Participant characteristics for circRNA expression in peripheral blood

**A.**					
	***p*-value**	**Control**		**IGT**	
		**Mean**	**SD**	**Mean**	**SD**
**Age**	0.016	52.45	17.08	59.26	14.31
**BMI**	< 0.001	26.51	4.13	28.93	3.68
**HbA1c**	–	5.61	0.34	–	–
**Glucose**	–	4.85	0.40	–	–
**Sex**	0.004	F (60%);	M (40%)	F (40%)	M (60%)
**Ethnicity**	0.611	white (99%)	other (1%)	white (100%)	other (0%)
**B.**					
	***p*-value**	**Control**		**T2D**	
		**Mean**	**SD**	**Mean**	**SD**
**Age**	< 0.001	52.45	17.08	68.74	10.65
**BMI**	< 0.001	26.51	4.13	30.61	5.99
**HbA1c**	–	5.61	0.34	–	–
**Glucose**	–	4.85	0.40	–	–
**Sex**	0.001	F (60%);	M (40%)	F (57%)	M (43%)
**Ethnicity**	0.445	white (99%)	other (1%)	white (99%)	other (1%)

A. Anthropometric characteristics of peripheral blood donors with normal blood glucose (*n* = 133) and those with impaired glucose tolerance (IGT; *n* = 46) B. Anthropometric characteristics of peripheral blood donors with normal blood glucose (*n* = 133) and those with overt T2D (*n* = 106). Differences in parameters between islet groups was determined by t-test

### Associations between peripheral blood circRNA expression and fasting glucose, HbA1c or IGT/T2D status

Peripheral blood RNA samples underwent cDNA synthesis using the EvoScript system and Universal cDNA Master kit (Roche Life Science, Burgess Hill, UK). Samples were normalised to 100 ng/ μL RNA prior to reverse transcription. Reactions were executed according to the manufacturer’s instructions, with a small amendment to extend the final 65 °C incubation to 30 min. We then assessed the expression of circRNAs that associated with T2D in islets donors by RT-qPCR as described above. Target abundance was assessed using the Comparative Ct method, and expressed relative to the geometric mean of the target and control set as a whole, since endogenous controls alone did not provide a robust baseline. Levels of each circRNA were then normalised to median levels in non-diabetic blood samples. Differential expression by diabetic status (no diabetes or IGT, IGT, overt diabetes) was then assessed by one way ANOVA using StataSE15 (StataCorp, Texas, USA) with adjustment made for potential confounders age, sex, BMI and ethnicity.

## Results

### CircRNA profiling in islets

**Fig. 1:**
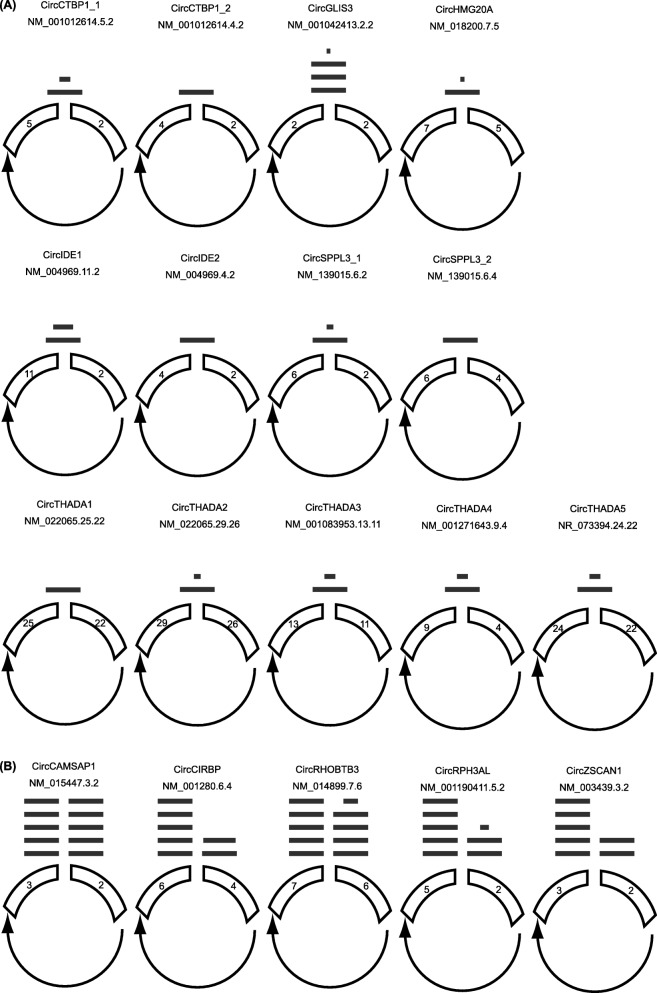
Structure of islet circRNAs: CirRNA junction schematics for 13 circular RNAs associated with Type 2 diabetes genome-wide association studies (**a**) and the 5 most abundant circRNAs identified in islets (**b**). Each schematic shows the identified backspliced exon or exons. The relative read depth at each backspliced junction is shown by the number of bars above each junction and is scaled by linear interpolation, where the backspliced junctions with 1 and 10 bars represent the junctions with the lowest and highest read depth respectively

Two thousand six hundred nineteen circRNAs were expressed in islet donors in the present study (Supplementary Table S3). Fourty-seven circRNAs had not been previously identified in data from multiple human tissues (multiple brain regions, muscle, thyroid and liver), and multiple cell types (including stem cells, skin and lung fibroblasts, neurons, lung epithelia, hepatocytes, breast cancer cells, lymphocytes, muscle myoblasts, aortic and vascular endothelial cells) analysed using the circBase and circAtlas databases [25] (http://zhaolab.biols.ac.cn/). These circRNA are given in Supplementary Table S4. The five circRNAs demonstrating the highest expression in human islets derived from the *CAMSAP1, CIRBP, RPH3AL, RHOBTB3* and *ZKSCAN1* loci. Thirteen circRNAs co-localized with the GWAS association signals for T2; these comprised *GLIS3* and *HMG20A* (1 circRNA each), *CTBP1, IDE and SPPL3* (2 circRNAs each) and *THADA* (five circRNAs). We selected these 18 circRNAs for further follow up. circRNA structures were predicted based on the sequencing read depth for each exon and are presented in Fig. 1. Exon structures presented as read depth plots are given in Supplementary Figure S1.

### Pathway analyses for genes generating islet-specific or abundant circRNAs

Pathway enrichment analysis was carried out to determine whether the genes hosting the 47 circRNAs not identified in other tissues were enriched in any specific gene ontology (GO) pathways. A similar analysis was also carried out the genes hosting the top 10% most abundant circRNAs. Pathways analysis was performed using ClueGO in Cytoscape (Bindea et al. 2009). The novel circRNAs demonstrated enrichment for genes in the ‘pancreatic secretion’ pathway (*p* = < 0.001). The 10% most abundantly expressed circRNAs were derived from genes demonstrating enrichment in the lysine degradation (*p* = 0.03), attenuation phase (*p* = 0.02), RUNX3 (*p* = 0.02), carcinoma (*p* = 0.01) and stem cell gene regulation (*p* < 0.001) pathways (Table 3).

**Table 3:** Pathways enriched in genes generating circRNAs expressed predominantly in human pancreatic islets

**A.**			
**Pathways enriched in genes hosting circRNAs not seen in other tissues**			
**Pathway**	***p*-value**	**Number of Genes**	**Genes**
Pancreatic secretion	< 0.0001	5	*CELA3A, CFTR, CPA1, KCNQ1, PNLIPRP2*
**B.**			
**Pathways enriched in genes generating the top 10% the most abundantly-expressed circRNAs in islets**			
**Pathway**	***p*-value**	**Number of Genes**	**Genes**
Hematopoietic Stem Cell Gene Regulation	< 0.0001	4	*CREBBP, EP300, FOXO3, GABPB1*
Pathways affected in Adenoid Cystic Carcinoma	0.010	6	*CREBBP,EP300, FOXO3, KANSL1, KMT2C, MAML3*
Attenuation phase	0.020	3	*CREBBP, DNAJB1, EP300*
RUNX3 regulates NOTCH signaling	0.020	3	*CREBBP, EP300, MAML3*
Lysine degradation	0.030	5	*ASHIL, EHMT1, KMT2C, PLOD1, SETD3*

GO pathways analysis to identify pathways enriched in the host genes of A. novel RNAs not previously characterised in other tissues or B. the top 10% most abundant circRNAs in human islets are presented here. Number of genes = number of differentially-expressed genes in each pathway

### CircRNAs are differentially expressed in a tissue-specific pattern

We assessed the expression patterns of the 18 circRNAs selected for further analysis across a panel of human tissues. We demonstrated that the expression patterns of circRNAs did not always correlate with levels of their corresponding linear transcripts. The expression levels of circular and linear forms of the gene were sometimes divergent, indicating that the circRNAs are regulated independently from the mRNAs also deriving from the parental linear gene (Supplementary Figure S2). For instance while *circSPPL3_2* was upregulated in most tissues compared to its linear gene, both *circCAMSAP1* and *circRHOBTB3* roughly followed the pattern of expression of their linear transcript levels across divergent tissues.

### The most abundant islet circRNAs are associated with insulin secretory index (SI) or T2D status in human islets

**Fig. 2:**
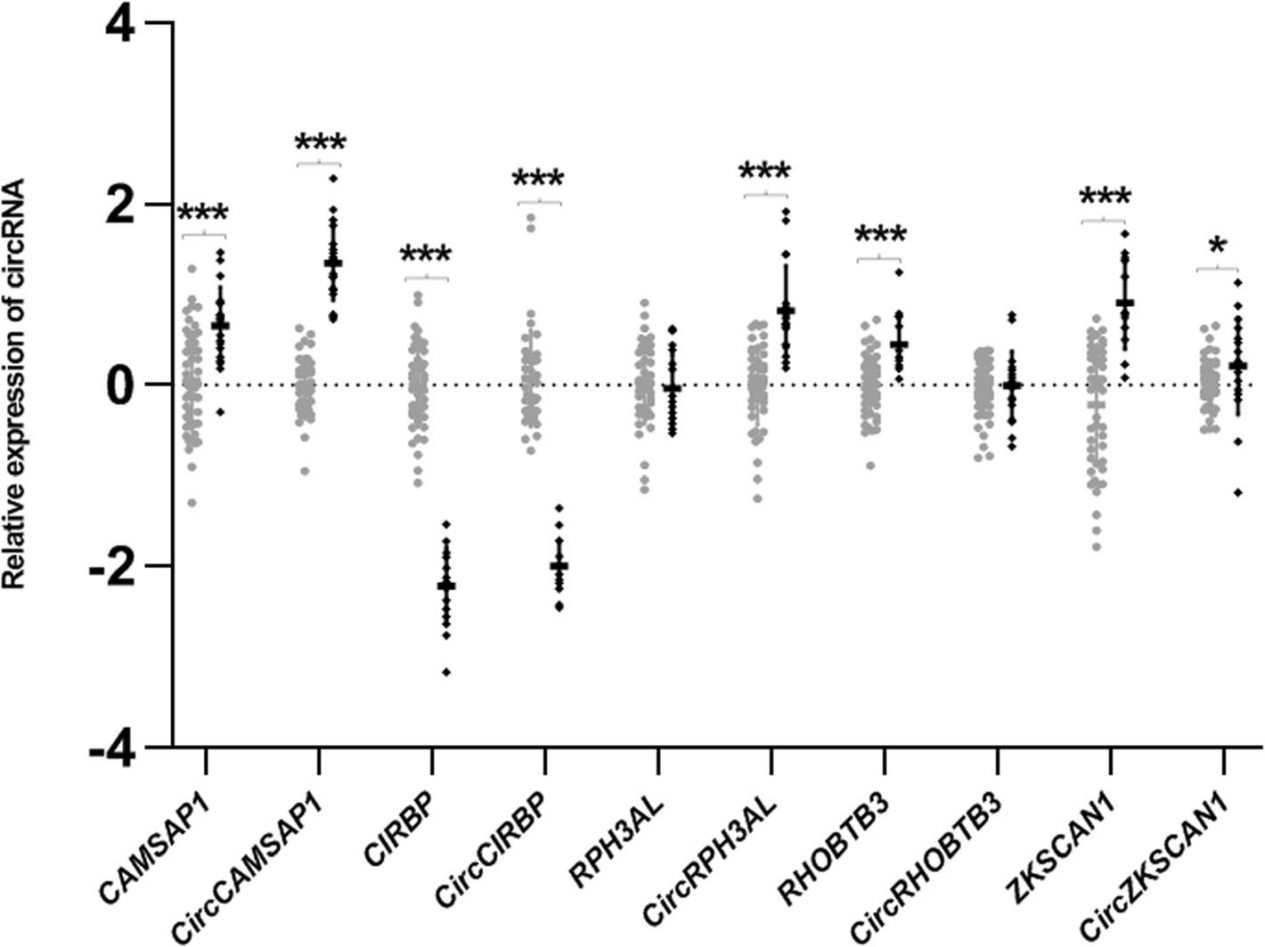
Differential expression of *circCAMSAP1, circCIRBP, circRPH3AL* and *circZKSCAN1* in diabetic islets. Expression levels of circular and linear transcripts of the top 5 most abundant circRNAs in human islets are given here in relation to T2D status. Islets from non-diabetic individuals are given in grey (*n* = 50), those from individuals with T2D are given in black (*n* = 20). Relative expression of each linear or circular RNA is given on the Y axis, and was quantified relative to the geometric mean across all samples, since endogenous control genes alone did not provide a stable baseline. Statistical significance in difference of expression between islets from donors with or without T2D is indicated by stars. * = < 0.05, ** = < 0.005, *** = < 0.001

4/5 circRNAs that showed marked expression in the islets demonstrated an association with T2D status (Fig. 2, supplementary Table S5). Three of these circRNAs, *circCAMSAP1*, *circCIRBP* and *circRPH3AL* satisfied the multiple testing threshold (*p* < 0.001). *CircZKSCAN1* showed nominal association with T2D status in islet donors (*p* = 0.030). Of these, the expression of the linear transcripts of three of these circRNAs, *CAMSAP1, CIRBP* and *ZKSCAN1* were also significantly associated with diabetic status (*p* < 0.001). *RHOBTB3* (*p* < 0.001) also demonstrated significant association with T2D status although its circRNA showed no association. The majority of these were positive associations, with the exception of *CIRBP* and *circCIRBP*, which were negatively correlated with T2D status. In addition, *circCIRBP* demonstrated a nominal negative association with insulin secretory index (*p* = 0.028; supplementary Table S5). No associations were identified between islet circRNA expression and donor HbA1c.

### CircRNA expression is not driven by genotype

We next assessed the expression of circRNAs located in regions of the genome linked to risk of T2D. Thirteen circRNAs co-localised with the genomic regions encompassing the GWAS association signals for T2D; 2 circRNAs from the *CTBP1* gene (in rs6819243 region), one circRNA from the *GLIS3* gene (rs10758593), one circRNA from the *HMG20A* gene (rs7177055), two circRNAs from the *IDE* gene (rs1111875), two circRNAs from the *SPPL3* gene (rs12427353) and five circRNAs deriving from the *THADA* gene (rs10203174). We identified no associations between any of these circRNAs and genotype at these loci (Table 4).

**Table 4:** Association of expression of circRNAs mapping to T2D-GWAS loci and their parental transcripts with genotype in primary non-diabetic islets

**Transcript**		**GG**		**Gg and gg**	
	***p*-value**	**Mean**	**SD**	**Mean**	**SD**
**rs6819243**					
*CTBP1*	0.781	−0.11	0.32	−0.04	0.34
*CircCTBP1_1*	0.274	−0.03	0.65	0.09	0.37
*CircCTBP1_2*	0.293	0.00	0.85	0.11	0.85
**rs10758593**					
*GLIS3*	0.139	−0.19	0.37	0.02	0.36
*CircGLIS3*	0.535	0.03	0.37	0.04	0.41
**rs7177055**					
*HMG20A*	0.502	−0.03	0.38	0.09	0.37
*CircHMG20A*	0.952	0.03	0.36	0.03	0.38
**rs1111875**					
*IDE*	0.578	−0.66	0.39	−0.46	0.35
*CircIDE1*	0.953	−0.02	0.40	0.04	0.45
*CircIDE2*	0.937	−0.02	0.23	0.02	0.36
**rs12427353**					
*SPPL3*	0.808	−0.01	0.48	0.05	0.43
*CircSPPL3_1*	0.677	−0.02	0.45	0.06	0.34
*CircSPPL3_2*	0.595	0.01	0.48	0.21	0.48
**rs10203174**					
*THADA*	0.369	−0.02	0.61	0.22	0.50
*CircTHADA1*	0.672	0.05	0.40	0.10	0.42
*CircTHADA2*	0.282	−0.01	0.86	− 0.33	0.96
*CircTHADA3*	0.133	0.11	0.32	0.16	0.45
*CircTHADA4*	0.319	0.06	0.39	−0.24	0.46
*CircTHADA5*	0.651	0.14	0.59	0.27	0.40

The association between circRNA/linear transcript expression and genotype for circRNAs located to GWAS loci for T2D are given. Heterozygous samples and minor allele homozygotes are combined into one category. SD = standard deviation. Genotypes: GG = most common allele observed. Gg = heterozygotes, gg = minor allele heterozygotes

### CircRNAs are differentially expressed upon exposure to stress conditions in EndoC-βH1 cells

Although the 5 most abundant circRNAs expressed in human islets did not show overt responsiveness to altered glucose, hypoxia or pro-inflammatory cytokines when tested in the human beta cell line EndoC-βH1, two (*circCIRBP* and *circRPHAL3*) did demonstrate changes in expression following treatment with 0.5 mM palmitate. *CircCIRBP* expression was increased following treatment (*P* = 0.021) whereas *circRPHAL3* demonstrated reduced expression (*p* = 0.022; Table 5). The expression of the linear transcripts from the *RHOBTB3* and *ZKSCAN1* genes also demonstrated increased expression in EndoC-βH1 cells treated with palmitic acid, in the absence of effects of their respective circRNAs.

**Table 5:** Expression of most abundantly expressed circRNAs and their parental transcripts in EndoC-βH1 cells treated with diabetes-related stresses

**Transcript**	**Treatment**	***p*-value**	**Median (IQR)**
*CAMSAP1*	Control		1.11 (0.95–1.39)
	2.5 mM glucose	0.313	1.03 (0.92–1.05)
	25 mM glucose	0.444	0.96 (0.95–1.17)
	Control		0.92 (0.89–1.06)
	1% O_2_	0.694	0.97 (0.73–1.04)
	3% O_2_	0.347	0.88 (0.63–0.99)
	Control		1.05 (0.95–1.08)
	Palmitic acid	0.062	0.89 (0.77–0.94)
	Control		0.93 (0.25–1.21)
	Cytokines	0.635	0.95 (0.90–0.99)
*CircCAMSAP1*	Control		0.84 (0.79–1.11)
	2.5 mM glucose	0.762	1.02 (0.75–1.12)
	25 mM glucose	0.681	0.92 (0.78–1.29)
	Control		0.98 (0.92–1.14)
	1% O_2_	0.136	0.89 (0.76–0.93)
	3% O_2_	0.292	0.83 (0.63–1.07)
	Control		1.04 (1.02–1.23)
	Palmitic acid	0.090	0.82 (0.65–1.00)
	Control		0.77 (0.27–1.14)
	Cytokines	0.555	0.99 (0.69–1.02)
*CIRBP*	Control		1.06 (0.83–1.08)
	2.5 mM glucose	0.080	1.38 (1.10–1.53)
	25 mM glucose	0.077	1.30 (1.14–1.56)
	Control		1.01 (0.99–1.09)
	1% O_2_	0.146	0.79 (0.39–0.97)
	3% O_2_	0.129	0.84 (0.74–1.02)
	Control		0.88 (0.65–1.09)
	Palmitic acid	0.907	0.91 (0.64–1.15)
	Control		0.90 (0.03–1.34)
	Cytokines	0.593	0.95 (0.93–1.06)
*CircCIRBP*	Control		0.77 (0.61–1.42)
	2.5 mM glucose	0.844	0.86 (0.32–1.37)
	25 mM glucose	0.652	0.77 (0.72–0.94)
	Control		1.10 (1.00–1.23)
	1% O_2_	0.955	0.86 (0.56–2.00)
	3% O_2_	0.726	0.89 (0.68–1.48)
	Control		0.75 (0.72–1.04)
	***Palmitic acid***	***0.021***	***1.69 (1.30–1.98)***
	Control		1.17 (1.00–1.33)
	Cytokines	0.180	0.73 (0.69–1.06)
*RPH3AL*	Control		1.05 (0.93–1.06)
	2.5 mM glucose	0.503	0.99 (0.75–1.07)
	25 mM glucose	0.441	0.93 (0.80–1.08)
	Control		1.09 (1.06–1.37)
	1% O_2_	0.357	1.55 (1.04–1.56)
	3% O_2_	0.205	1.32 (1.28–1.81)
	Control		0.95 (0.87–0.99)
	Palmitic acid	0.102	0.84 (0.70–0.87)
	Control		0.98 (0.02–1.01)
	Cytokines	0.354	1.02 (0.91–1.12)
*CircRPH3AL*	Control		1.00 (0.45–1.00)
	2.5 mM glucose	0.941	0.85 (0.58–0.97)
	25 mM glucose	0.867	0.76 (0.73–0.86)
	Control		1.09 (1.06–1.37)
	1% O_2_	0.357	1.55 (1.04–1.56)
	3% O_2_	0.205	1.32 (1.28–1.81)
	Control		1.66 (1.32–1.85)
	***Palmitic acid***	***0.022***	***0.97 (0.95–1.11)***
	Control		0.68 (0.47–0.77)
	Cytokines	0.295	0.97 (0.55–1.02)
*RHOBTB3*	Control		1.24 (1.23–1.31)
	2.5 mM glucose	0.188	1.11 (1.00–1.27)
	25 mM glucose	0.117	1.20 (0.72–1.14)
	Control		0.95 (0.69–1.06)
	1% O_2_	0.949	0.97 (0.77–0.98)
	3% O_2_	0.353	0.67 (0.65–0.93)
	Control		0.80 (0.72–0.90)
	**Palmitic acid**	**0.003**	**1.18 (1.15–1.21)**
	Control		1.17 (0.29–1.35)
	Cytokines	0.186	1.59 (1.22–1.72)
*CircRHOBTB3*	Control		0.74 (0.73–1.00)
	2.5 mM glucose	0.836	0.89 (0.65–1.02)
	25 mM glucose	0.351	0.77 (0.57–0.78)
	Control		1.04 (0.72–1.08)
	1% O_2_	0.182	0.78 (0.74–0.78)
	3% O_2_	0.288	0.79 (0.77–0.86)
	Control		1.16 (0.99–1.27)
	Palmitic acid	0.306	1.03 (0.83–1.13)
	Control		1.01 (0.95–1.08)
	Cytokines	0.628	0.97 (0.89–1.06)
*ZKSCAN1*	Control		1.24 (1.18–1.33)
	2.5 mM glucose	0.632	1.23 (1.05–1.87)
	25 mM glucose	0.997	1.23 (1.16–1.36)
	Control		0.93 (0.86–1.08)
	1% O_2_	0.388	1.06 (0.94–1.12)
	3% O_2_	0.218	0.72 (0.66–0.98)
	Control		0.50 (0.39–0.55)
	**Palmitic acid**	**0.001**	**1.01 (0.94–1.12)**
	Control		1.29 (0.24–1.33)
	Cytokines	0.378	1.30 (1.27–1.35)
*CircZKSCAN1*	Control		0.86 (0.81–1.17)
	2.5 mM glucose	0.490	0.91 (0.83–1.17)
	25 mM glucose	0.505	0.88 (0.83–1.28)
	Control		1.11 (0.99–1.42)
	1% O_2_	0.060	1.65 (1.41–1.79)
	3% O_2_	0.631	1.24 (1.02–1.57)
	Control		1.01 (0.81–1.45)
	Palmitic acid	0.252	0.83 (0.75–0.91)
	Control		1.05 (0.10–1.13)
	Cytokines	0.292	1.16 (1.13–1.18)

We assessed the effect of diabetes-related cellular stresses (low/high glucose, elevated fatty acid, hypoxia and exposure to pro-inflammatory cytokines) on the expression of the 5 most abundant islet circRNAs in the Endo-βH1 human beta cell line. IQR = interquartile range. Results meeting the threshold for 4 test conditions are given in bold underlined type, those presenting nominal associations only are indicated in bold italic type

### CircCAMSAP1 is differentially expressed in T2D peripheral blood

**Fig. 3:**
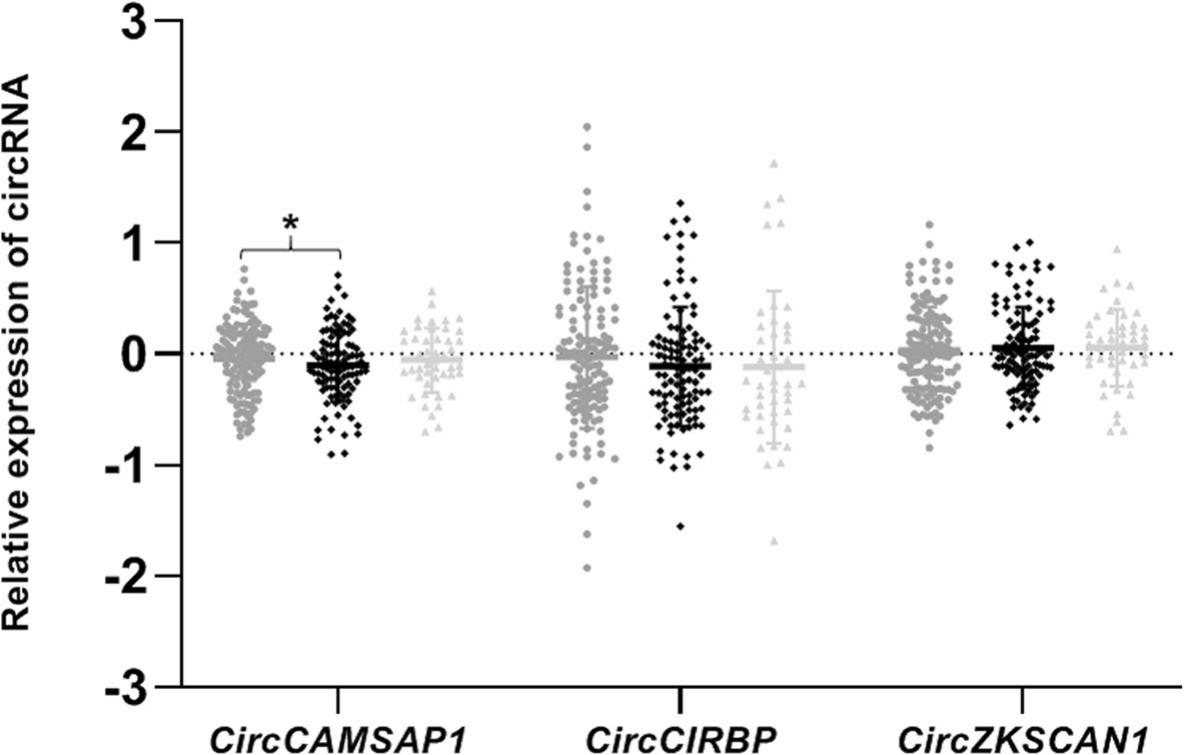
Differential expression of *circZKSCAN1* in peripheral blood of T2D participants Peripheral blood circRNA levels are given here for non-diabetic samples (labelled in mid-grey; *n* = 133), samples from individuals who have overt diabetes (labelled in black; *n* = 106) and those with impaired glucose tolerance IGT (labelled in light grey; *n* = 46). Relative expression of circRNAs is given on the Y axis and was quantified relative to the geometric mean across all samples, since endogenous control genes alone did not provide a stable baseline. Statistical significance in difference of expression between early and late passage cells is indicated by stars. * = < 0.05, ** = < 0.005, *** = < 0.001

Of the 4 circRNAs demonstrating evidence of altered expression in the islets of donors with T2D, two (*circIDE1* and *circRPH3AL)* were not expressed in peripheral blood. Of the three remaining circRNAs with expression in peripheral blood (*circCAMSAP1*, *circCIRBP* and *circZKSCAN1*), *circCAMSAP1* demonstrated a nominal negative association with diabetes status in the peripheral blood of patients with T2D (Fig. 3; supplementary Table S6). No difference in expression was detected for any circRNA between control patients and those with IGT, or between those with IGT and those with T2D). No associations were evident between circRNA expression in peripheral blood and either participant fasting glucose or HbA1c.

## Discussion

We present here the first enriched circRNA profile from human primary pancreatic islet RNA produced from a modified NGS CircleSeq protocol with enrichment for circRNAs. We have identified 2619 circRNAs expressed in human islets, including 47 circRNAs which were not identified in profiles from multiple other tissues in circBase or circAtlas. Some of these novel circRNA are clustered into pathways representative of pancreatic exocrine function, so it is possible that their expression is pancreatic, rather than islet specific. Of the 18 circRNAs selected for follow up on the basis of abundance or co-localisation to the GWAS association signals for T2D, many also show evidence of regulation independent of their parent gene. Four out of 5 of the most abundant circRNAs in human islets demonstrate strong evidence of dysregulated expression in the islets of human donors with T2D, with one (*circCIRBP*) demonstrating an association with insulin secretory index. Two out of 5 also show dysregulated expression in human EndoC-βH1 beta cells treated with fatty acids although direction of effect was not conserved. Finally, one circRNA (*circCAMSAP1*) also demonstrates an association with diabetes status in the peripheral blood of patients with type 2 diabetes.

To date, there have been two circRNA profiles generated from human pancreatic endocrine cells or intact islets. The first provides a circRNA profile generated from publically-available NGS data from isolated α, β and δ cell transcriptomes [17]. This study reported 10,832 putative circRNAs expressed in total, with 382 shared across cell types. This study reports more islet circRNAs than identified in our dataset, but this profile is derived from conventional NGS data, with no pre-treatment to remove linear sequences. Back splicing events can be generated from tandem DNA duplications within genes, or from trans-splicing events during linear splicing [26], so it is likely that profiles derived from conventional NGS contain sequences that in fact represent aberrantly spliced linear transcripts rather than genuine circRNAs. Differences will also arise in that this previous circRNA profile derives from isolated α, β and δ cell populations, whereas ours is a profile derived from intact islets. Differences in gene expression patterns may thus reflect the effects of cell:cell crosstalk as occurs in vivo. Nevertheless, there was considerable overlap between our profile and the beta cell circRNA profile of Kaur et al. [17]. Of the top 100 most abundant circRNA in the Kaur profile, all but 12 had counterparts from the same genes in our profile. Clustering between the most abundant genes in the Kaur profile and the most abundant genes in the profile reported here was also apparent (supplementary Table S3).

A second islet circRNA profile has also been reported [18]. This profile was based on a microarray approach, identified 3441 islet circRNAs from a study of 3 human islet samples (two female and one male donor). Since this is a microarray-based profile, it will only contain circRNAs that have been already annotated. The most abundant circRNA in this study derived from the *HIPK3* gene. We also identified a circRNA deriving from this gene in the top 75 most abundant circRNA transcripts in our profile. This study differs from ours in that follow up work on circRNA expression in cell lines and in relation to T2D status occurred in animal models and not in human cells and tissues.

Our data, like those reported in previous islet studies [17, 18] suggests that many of the circRNAs we have identified are regulated independently of their linear counterparts (Supplementary Figure S2). In some cases, we have identified associations between cell treatments or T2D status with circRNAs in the absence of apparent effects on their linear transcripts. Comparison of circRNA expression across tissues showed expression patterns of many circRNAs were often higher in brain tissues compared to other tissues, which is in line with existing knowledge that circRNAs accumulate in the brain [27].

Some of our circRNAs are associated with glycaemic traits in human islets. The expression of *circCIRBP* demonstrates a negative association with insulin secretory index (SI) of the donor islets, is elevated in human EndoC-βH1 beta cells treated with palmitic acid and is reduced in islets from donors with T2D. The parent gene *CIRBP* (Cold Inducible RNA Binding Protein) has roles in genotoxic stress response, not only from cold, but also from other cellular stressors such as hypoxia [28]. Elevated levels have been associated with maintenance of glucose metabolism and protection from cold exposure through effects on the AKT pathway [29]. The elevated levels we demonstrate in the human beta cell line EndoC-βH1 treated with palmitate may therefore represent an acute stress response to altered lipid. The lower levels in the islets of individuals with T2D may reflect lower stress tolerance in diabetic islets, and as may the inverse correlation with SI, since we have previously demonstrated that stressed beta cells can transdifferentiate into delta cells in vitro [23].

We also identified elevated levels of both *circCAMPSAP1* and its host gene *CAMSAP1* in the islets of donors with T2D. *CAMSAP1* encodes an organisation protein involved in microtubule dynamics and localisation [30]. The dynamics of microtubule assembly and disassembly has an impact on the insulin secretion machinery; translocation and movement of insulin granules along microtubules can influence their availability for secretion. Failure to disassemble can impede docking. Microtubule density is higher in the islets of diabetic mice compared with non-diabetic littermates [31]. We also identified an association between *circCAMSAP1* expression and diabetes status in the peripheral blood of individuals with T2D, although this was the inverse of that seen in islets. CAMSAPs are active in multiple tissues, and also have roles in white blood cells, which rely on the tubulin-microtubule system for lymphocyte activation [32]. The effect of T2D status on *circCAMSAP1* expression in blood may therefore reflect potential tissue-specific activities of this circRNA. *ZKSCAN1* and its circular RNA *circZKSCAN1* have been described as inhibitors of cellular proliferation and survival [33]. Both transcripts demonstrate elevated expression in islets from donors with T2D, which may perhaps reflect adverse effects on beta cell survival. *CircRPH3AL* was also upregulated in diabetic islets. Linear transcripts from *RPH3AL* are highly expressed in β-cells and have roles in calcium-dependent exocytosis during granule maturation and insulin secretion [34].

We hypothesised that dysregulation of circRNA expression may underpin some of the GWAS association signals for T2D. Thirteen circRNAs colocalised to the recombination windows surrounding the 6 of the GWAS index loci, but none of these demonstrated differential expression by risk genotype. This suggests that the genetic associations between individual genetic variants and T2D is probably not mediated by dysregulation of islet circRNAs.

We acknowledge that at present however, our data are largely correlative, and at present do not offer information on causality, definitive mechanistic proof or insight into the regulatory relationships between circRNAs and their host genes. CircRNAs can have effects *in cis* by regulating the transcription, linear splicing or translation of linear transcripts from their host genes [35–40]. In our data, we observe similar responses of linear and circular transcripts in response to challenge (*CAMSAP1/circCAMSAP1*, *CIRBP/circCIRBP, ZKSCAN1/circZKSCAN1*). This may be a manifestation of effects on transcription of common pre-RNAs from which both forms can be expressed. Other circRNAs show dysregulated expression for the circRNA alone (*circRPH3AL*). This suggests that the effect of circRNA regulation is post-transcriptional in these cases. CircRNAs can also act *in trans*, by virtue of sponging of other non-coding (nc) RNAs or RNA binding proteins [41–43]. In these cases, it is impossible to deduce from our data what the molecular targets of dysregulated islet circRNAs may be.

## Conclusion

In conclusion, we present here an enriched circular RNA profile in human pancreatic islets. Although we find no evidence that the associations between T2D and genetic variation are underpinned by effects on the circRNA milieu, we demonstrate that the majority of the most abundant islet circRNAs display associations between their expression and aspects of glucose homeostasis in human donors, as well as associations with other glycaemic measures. One circRNA, *circCAMSAP1*, also demonstrated altered expression in the peripheral blood of individuals with T2D, and may have future utility as a biomarker.

## Supplementary information

**Additional file 1.**

**Additional file 2.**

**Additional file 3.**

**Additional file 4.**

**Additional file 5.**

**Additional file 6.**

**Additional file 7.**

**Additional file 8.**

## Data Availability

All data generated or analysed during this study are included in this published article and its supplementary information files. Raw reads are deposited in the Sequence Read Archive (SRA) BioProject database using BioProject ID PRJNA607015 (https://www.ncbi.nlm.nih.gov/bioproject/?term=PRJNA607015). The raw reads can be downloaded via their SRR IDs for the mock-treated (SRR11095576) and RNAse R treated samples (SRR11095575). CircRNA data from isolated islet cell subtypes was accessed through the supplementary information given in [17].

## Abbreviations

T2D: Type 2 diabetes
GWAS: Genome wide association study
ncRNA: Non-coding RNA
miRNA: MicroRNA
NGS: Next generation sequencing
RIN: RNA integrity number
qRT-PCR: Quantitative reverse transcription PCR
WGA: Whole Genome amplification
cDNA: Complementary DNA
TNFa: Tumour necrosis factor alpha
IFNg: Interferon gamma
IL-1b: Interleukin 1 beta
HbA1c: Glycosylated haemoglobin
IGT: Impaired glucose tolerance
SI: Insulin rsecretory index.
