# Supplementary figures and images for: Islet-expressed circular RNAs are associated with type 2 diabetes status in human primary islets and in peripheral blood
Source: BMC Med Genomics. 2020 Apr 20;13:64. doi: 10.1186/s12920-020-0713-2 (PMC7171860; doi:10.1186/s12920-020-0713-2)

(A)

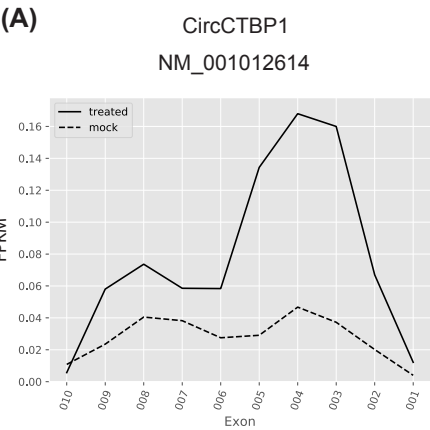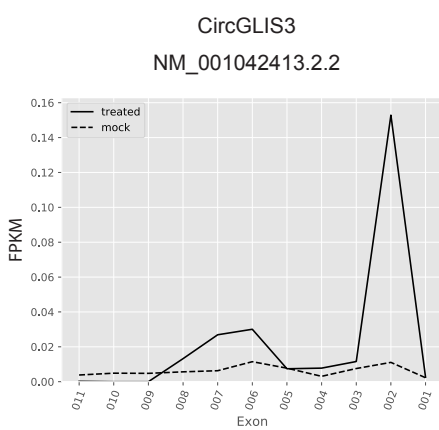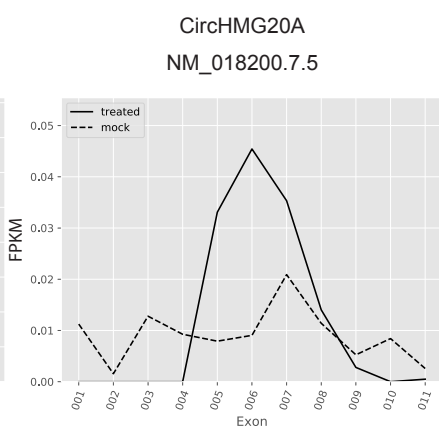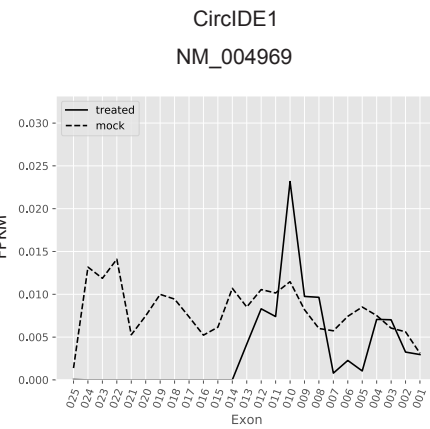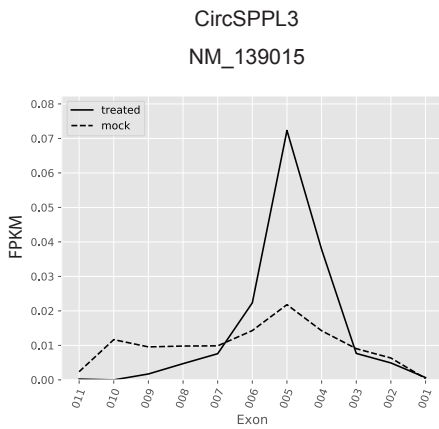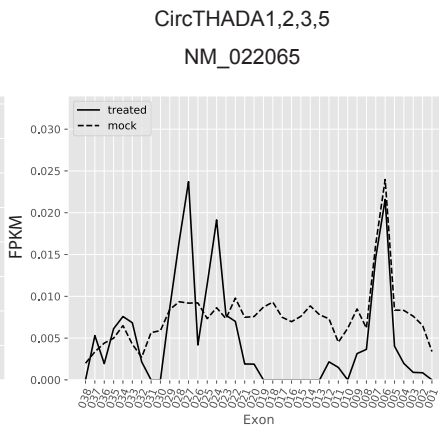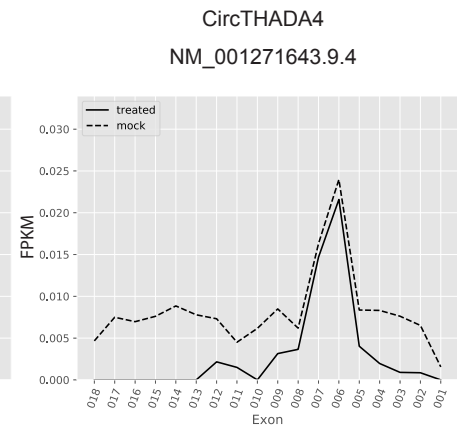

(B)

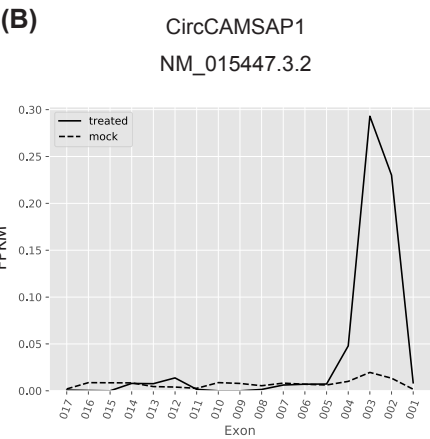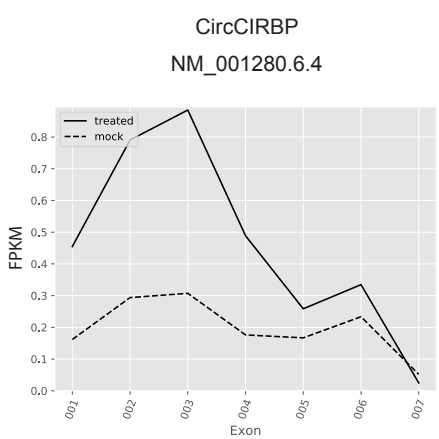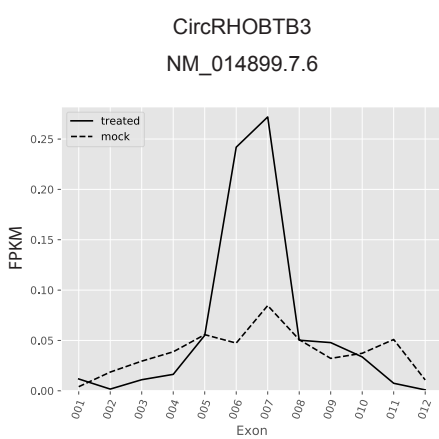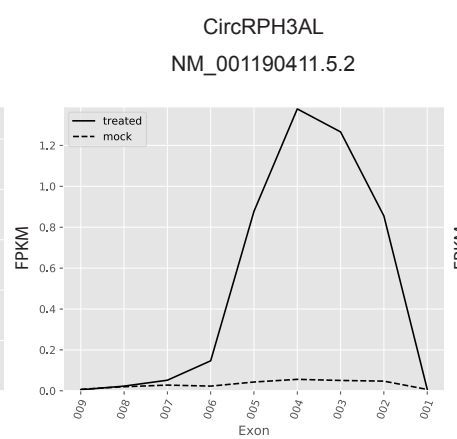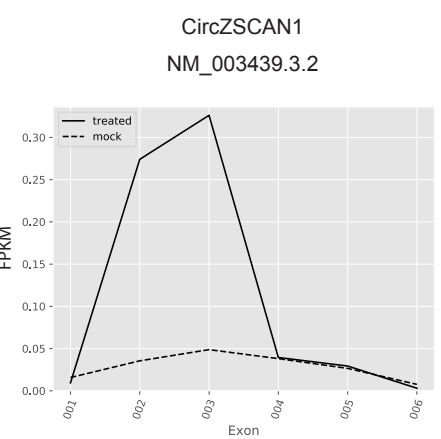

Supplement: Supplementary file 1 — Additional file 1. [file 12920_2020_713_MOESM1_ESM.pdf]
