# Supplementary material for: Islet-expressed circular RNAs are associated with type 2 diabetes status in human primary islets and in peripheral blood
Source: BMC Med Genomics. 2020 Apr 20;13:64. doi: 10.1186/s12920-020-0713-2 (PMC7171860; doi:10.1186/s12920-020-0713-2)
Supplement: Supplementary file 2 — Additional file 2. [file 12920_2020_713_MOESM2_ESM.pdf]

A.

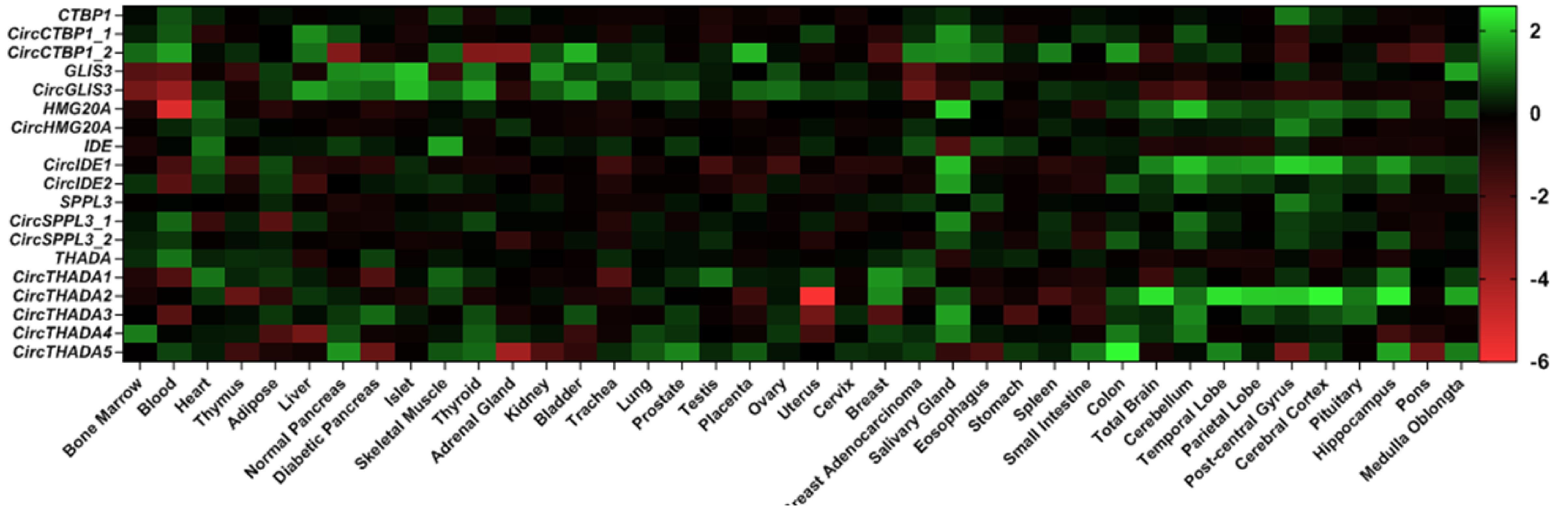

B.

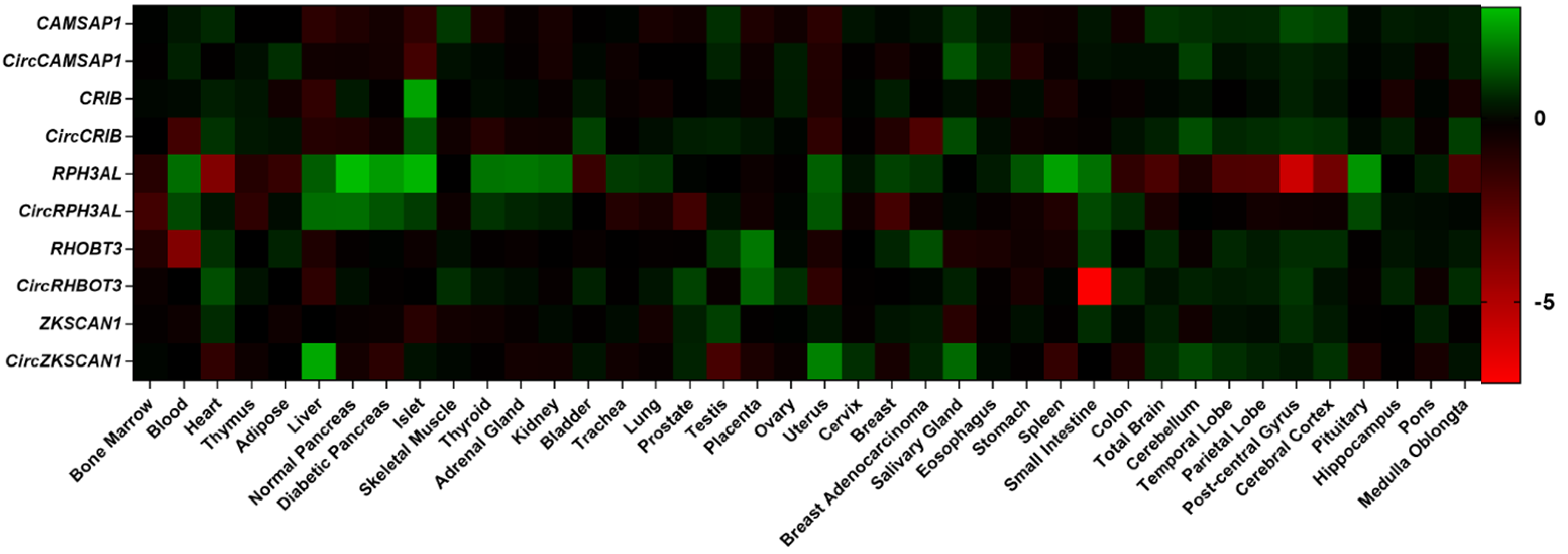

Supplementary figure S2: Tissue profile of islet and GWAS-located circRNAs Relative expression of circRNA and their cognate transcripts have been assessed in different tissue samples. The expression profile of 13 GWAS-localizing circRNAs are shown in **(A)** and 5 most abundant circRNAs are shown in **(B)** alongside their linear counterparts RNA.
