# Supplementary material for: Islet-expressed circular RNAs are associated with type 2 diabetes status in human primary islets and in peripheral blood
Source: BMC Med Genomics. 2020 Apr 20;13:64. doi: 10.1186/s12920-020-0713-2 (PMC7171860; doi:10.1186/s12920-020-0713-2)
Supplement: Supplementary file 3 — Additional file 3. [file 12920_2020_713_MOESM3_ESM.pdf]

Supplementary table S1: Details of individual islet preps uised in this study. The specifics of each islet prep used in this study are given below. '1' refers to islets used in the comparison of circRNA levels in relation to Type 2 diabetes (T2D) status. '2' refers to isles used in the correlation of circRNA expression with genotype at GWAS association signals. BMI = Body Mass index. SF = Snap Frozen, F = Fresh, IIDP-W = Wisconsin centre, IIDP-P = Pennsylvania centre, IIDP-I = Illinois centre, IIDP-SLI = Scharp Lacy institute, IIDP-M = Miami centre, CICRC - California Islet Cell Resources centre, AIX = AIX Biotech LLC, AMS = AMS Biosciences, NDRI = National Disease Research Interchange.

Islet identifier	Study	Age	Sex	BMI	Ethnicity	Diabetes status	Insulin release	HbA1c (%)	Shipped	Isolation Centre
AACD168A/UWHI222R	1,2	36	F	45	White	No	5.9	ND	SF	IIDP-W
ABHB331	1,2	43	M	37	White	No	2.1	ND	SF	IIDP-W
ACB0251/UWHI243R	1,2	45	F	33	White	No	2.3	ND	SF	IIDP-W
ACEA213-A/UWHI247R-A	1,2	53	F	21	White	No	1.4	5.5	SF	IIDP-W
ACFO053-A/UWHI248R-A	1,2	29	M	31	White	No	2.4	5.5	SF	IIDP-W
ACJY368/ICRH-86	2	42	F	23	White	No	ND	5.4	F	IIDP-P
H539/ACAF253	1,2	41	M	31	White	No	1.6	ND	SF	IIDP-I
H546/ACDF368	1,2	59	M	20	Afr Amer	No	1.3	5.5	SF	IIDP-I
H549/ACDW320	1,2	47	M	36	Afr Amer	No	0.9	ND	SF	IIDP-I
HP-15016-01A/ACAN292A	1	55	M	33	White	No	4.2	5.9	SF	IIDP-SLI
HP-15024-01/ACAV429	1	25	M	25	White	No	3.7	5.3	SF	IIDP-SLI
HP15175	1	21	M	24	White	No	1.6	5.6	SF	IIDP-SLI
HP-15157-01A/ACFC446A	2	27	M	19	White	No	1.6	5.8	SF	IIDP-SLI
HP1889	1,2	37	M	37	White	No	1	ND	SF	IIDP-M
HP1963	1,2	55	F	26	White	No	1.45	ND	SF	IIDP-M
HP1986	1,2	43	F	30	White	No	3.66	ND	SF	IIDP-M
HP1995	1,2	24	F	23	White	No	3.46	ND	SF	IIDP-M
HP2026	2	27	M	30	White	No	6.74	ND	SF	IIDP-M
HP2055	1,2	44	F	22	White	No	1.70	ND	SF	IIDP-M
HP2056	1,2	39	F	30	Afr Amer	No	3.53	ND	SF	IIDP-M
HP2062	1	20	M	26	White	No	1.10	ND	SF	IIDP-M
HP2072	1	55	M	29	White	No	2.44	ND	SF	IIDP-M
HP2075	1	57	F	25	White	No	4.64	ND	SF	IIDP-M
HP2076	1	48	M	22	White	No	4.59	ND	SF	IIDP-M

HP2079	2	56	F	26	White	No	ND	ND	SF	IIDP-M
HP2080	1,2	46	M	27	White	No	2.15	ND	SF	IIDP-M
HP2092	1	51	F	26	White	No	4.27	ND	SF	IIDP-M
HP2095	2	31	M	29	White	No	2.64	ND	SF	IIDP-M
HP2097	1,2	61	F	32	White	No	4.12	ND	SF	IIDP-M
HP2098	1,2	56	F	30	Hispanic	No	1.81	ND	SF	IIDP-M
HP2099	1,2	50	M	26	Hispanic	No	3.01	ND	SF	IIDP-M
HP2101	1,2	20	M	24	Hispanic	No	2.36	ND	SF	IIDP-M
HP2103	1,2	24	F	27	White	No	3.36	ND	SF	IIDP-M
HP2106	1,2	51	M	26	Afr Amer	No	4.07	ND	SF	IIDP-M
HP2116	1	53	M	29	Hispanic	No	2.23	ND	SF	IIDP-M
HP2148	1	20	M	21	White	No	1.5	ND	SF	IIDP-M
HP2182	1,2	24	F	35	White	No	3.7	ND	SF	IIDP-M
HP2186	1,2	19	M	20	White	No	1.4	ND	SF	IIDP-M
HP2187B/ACDL087B	2	42	M	37	Hispanic	No	7.2	5.3	SF	IIDP-M
HP2189	1,2	64	F	25	White	No	2.06	ND	SF	IIDP-M
HP2191A/ACEK077A	1,2	53	F	21	White	No	1.4	5.5	SF	IIDP-M
HP2197/ACGK380	1,2	45	F	23	White	No	5	5.5	SF	IIDP-M
HP2203/ACHU294	1,2	17	M	22	White	No	1.19	ND	SF	IIDP-M
HP2204/ACH4140	1,2	57	F	27	White	No	1.9	5.3	SF	IIDP-M
Hu779	2	52	F	25	White	No	ND	ND	SF	CICRC
Hu781	2	30	M	32	White	No	ND	ND	SF	CICRC
Hu782a	2	62	F	24	White	No	ND	ND	SF	CICRC
Hu932A/ACAM113A	1,2	18	M	28	White	No	1.8	5	SF	CICRC
Hu943/ACEC298	1,2	27	M	31	Afr Amer	No	0.9	5.4	SF	CICRC
Hu945/ACEU273	1,2	53	M	22	White	No	1.5	5.5	SF	CICRC
Hu949/ACFL177	1,2	39	M	29	White	No	1.2	5.6	SF	CICRC
Hu953/ACGX022	2	57	F	28	White	No	ND	5.5	SF	CICRC
OD34316	2	47	M	20	White	No	ND	ND	SF	AIX
OD36812	2	40	F	23	Afr Amer	No	ND	ND	SF	AIX
rHIP81	1,2	30	M	29	White	No	3.58	ND	SF	AIX
rHIP82	2	40	F	25	Asian	No	8.2	ND	SF	AIX
rHIP83	1,2	51	M	28	Hispanic	No	2.5	ND	SF	AIX
rHIP85	1,2	31	F	25	White	No	2	5.1	SF	AIX
rHIP87	1,2	59	M	30	White	No	1	ND	SF	AIX

rHIP90	1,2	32	M	44	Hispanic	No	2.53	6.3	SF	AIX
rHIP91	1,2	57	F	56	White	No	1.08	ND	SF	AIX
rHIP92	1,2	27	M	25	Filipino	No	1.3	4.6	SF	AIX
rHIP93	1,2	22	M	25	Asian	No	2.8	5.4	SF	AIX
ABKO130/Hu926	1	52	M	32	Hispanic	Yes	0.30	6.70	F	CICRC
XIX456/HP10269	1	43	M	27	Hispanic	Yes	ND	ND	SF	IIDP-SLI
HP-15085-01T2DA/ACCY386A	1	37	F	39	White	Yes	2.80	7.30	SF	IIDP-SLI
HP1991	1	67	M	30	White	Yes	1.54	ND	SF	IIDP-M
HP1997	1	58	M	24	White	Yes	ND	ND	SF	IIDP-M
HP2020	1	65	F	31	Afr Amer	Yes	1.32	ND	SF	IIDP-M
HP2029	1	48	F	23	Afr Amer	Yes	1.37	ND	SF	IIDP-M
HP2032	1	55	M	28	White	Yes	3.77	ND	SF	IIDP-M
HP2039	1	54	M	30	Hispanic	Yes	ND	ND	SF	IIDP-M
HP2043	1	65	M	33	White	Yes	ND	ND	SF	IIDP-M
HP2047	1	43	F	33	White	Yes	3.41	ND	SF	IIDP-M
HP2049	1	60	F	29	Afr Amer	Yes	ND	ND	SF	IIDP-M
HP2067	1	64	F	33	White	Yes	2.81	ND	SF	IIDP-M
HP2069	1	53	F	31	Afr Amer	Yes	5.23	ND	SF	IIDP-M
HP2071	1	43	M	37	Afr Amer	Yes	2.73	ND	SF	IIDP-M
HP2081	1	65	F	29	White	Yes	1.50	ND	SF	IIDP-M
HP2107	1	45	M	21	Afr Amer	Yes	9.16	ND	SF	IIDP-M
ILT092710	1	54	F	53	Afr Amer	Yes	ND	ND	SF	AMS
ILT1106901	1	42	M	32	White	Yes	ND	ND	SF	AMS
OD35464	1	58	M	66	Afr Amer	Yes	ND	ND	SF	NDRI
