# Supplementary material for: Islet-expressed circular RNAs are associated with type 2 diabetes status in human primary islets and in peripheral blood
Source: BMC Med Genomics. 2020 Apr 20;13:64. doi: 10.1186/s12920-020-0713-2 (PMC7171860; doi:10.1186/s12920-020-0713-2)
Supplement: Supplementary file 4 — Additional file 4. [file 12920_2020_713_MOESM4_ESM.docx]

**Supplementary table S2: CircRNA probe and primer sequences**

**Circular RNA probes**

**Linear RNA assays**

**Genotyping primers**
