# Supplementary material for: Islet-expressed circular RNAs are associated with type 2 diabetes status in human primary islets and in peripheral blood
Source: BMC Med Genomics. 2020 Apr 20;13:64. doi: 10.1186/s12920-020-0713-2 (PMC7171860; doi:10.1186/s12920-020-0713-2)
Supplement: Supplementary file 6 — Additional file 6. [file 12920_2020_713_MOESM6_ESM.pdf]

Supplementary table S4: Islet circRNAs not identified in other tissue types. These circRNAs have not been previously identified in data from multiple human tissues (multiple brain regions, muscle, thyroid and liver), and multiple cell types (including stem cells, skin and lung fibroblasts, neurons, lung epithelia, hepatocytes, breast cancer cells, lymphocytes, muscle myoblasts, aortic and vascular endothelial cells)

Backsplice junction	Gene ID	chromosome	start	stop	raw_count	BPM
NM_198850.9.5	PHLDB3	chr19	43998853.00	44002028.00	16	159.213802
NM_173812.16.12	DPY19L2	chr12	63987866.00	64002455.00	13	129.361214
NM_139025.22.20	ADAMTS13	chr9	136309983.00	136313849.00	13	129.361214
NM_017935.10.8	BANK1	chr4	102942670.00	102951422.00	12	119.410352
NR_023386.6.6	CROCCP3	chr1	16812920.00	16813156.00	11	109.459489
NR_026899.10.5	ARHGAP27P1-BPTFP1-KP	chr17	62750122.00	62758828.00	10	99.5086264
NM_005747.4.3	CELA3A	chr1	22331939.00	22332289.00	10	99.5086264
NM_052956.11.10	ACSM1	chr16	20636744.00	20638638.00	10	99.5086264
NM_001146320.4.2	GRAMD2B	chr5	125801117.00	125809060.00	9	89.5577638
NM_000818.16.4	GAD2	chr10	26507971.00	26589924.00	9	89.5577638
NM_182587.46.43	UNC80	chr2	210805944.00	210810667.00	9	89.5577638
NM_001040092.8.4	ENPP2	chr8	120628504.00	120633759.00	9	89.5577638
NM_017752.14.13	TBC1D8B	chrX	106096750.00	106097526.00	9	89.5577638
NM_003428.4.3	ZNF84	chrUn_gl000223	57230.00	58124.00	8	79.6069011
NM_001004431.2.2	METRNL	chr17	81042813.00	81043199.00	8	79.6069011
NM_005935.15.13	AFF1	chr4	88047250.00	88048855.00	8	79.6069011
NM_032504.6.2	UNC80	chr2	210637817.00	210654329.00	6	59.7051758
NM_007030.2.2	TPPP	chr5	677864.00	678179.00	6	59.7051758
NM_014760.3.3	TATDN2	chr3	10301820.00	10302354.00	6	59.7051758
NM_001004303.16.12	FYB2	chr1	57192169.00	57207894.00	6	59.7051758
NM_181798.6.3	KCNQ1	chr11	2591857.00	2594216.00	6	59.7051758
NR_003290.8.6	EP400P1	chr12	132604964.00	132606525.00	6	59.7051758
NM_001278250.3.3	SLC25A45	chr11	65146846.00	65147032.00	6	59.7051758
NM_173575.11.3	STK32C	chr10	134022537.00	134041632.00	4	39.8034506
NM_015082.13.9	FSTL4	chr5	132552920.00	132561511.00	4	39.8034506
NM_000352.4.2	ABCC8	chr11	17484984.00	17496574.00	4	39.8034506

NM_182911.19.17	TSGA10	chr2	99634662.00	99636945.00	4	39.8034506
NR_027300.16.15	C3P1	chr19	10165922.00	10166437.00	3	29.8525879
NM_138364.4.3	PRMT9	chr4	148594109.00	148595025.00	2	19.9017253
NM_033049.5.4	MUC13	chr3	124639080.00	124641150.00	2	19.9017253
NM_001868.4.3	CPA1	chr7	130021470.00	130022050.00	2	19.9017253
NM_001099409.5.5	EHBP1L1	chr11	65347551.00	65347730.00	2	19.9017253
NM_001161426.3.2	NUDT2	chr19	52850959.00	52852488.00	2	19.9017253
NM_194285.3.3	SPTY2D1	chr11	18636109.00	18637645.00	2	19.9017253
NM_032415.5.3	CARD11	chr7	2983845.00	2987421.00	2	19.9017253
NM_000492.11.4	CFTR	chr7	117170952.00	117199709.00	2	19.9017253
NM_005396.6.4	PNLIPRP2	chr10	118385456.00	118387385.00	1	9.95086264
NM_001202425.3.2	ZNF559-ZNF177	chr19	9475537.00	9482428.00	1	9.95086264
NM_014907.9.6	FRMPD1	chr9	37719065.00	37731100.00	1	9.95086264
NR_027460.4.2	RRN3P3	chr16	22444046.00	22447133.00	1	9.95086264
NR_029410.5.4	LOC389765	chr9	88444377.00	88445491.00	1	9.95086264
NM_173481.3.2	MISP	chr19	756889.00	760039.00	1	9.95086264
NM_031481.7.3	SLC25A18	chr22	18062510.00	18066300.00	1	9.95086264
NM_005400.13.12	PRKCE	chr2	46372231.00	46378368.00	1	9.95086264
NM_001278722.5.2	IL20RA	chr6	137329735.00	137338240.00	1	9.95086264
NM_001127384.5.2	CTNNA3	chr10	69281599.00	69407276.00	1	9.95086264
NM_005656.5.4	TMPRSS2	chr21	42860320.00	42861520.00	1	9.95086264
