# Supplementary material for: Islet-expressed circular RNAs are associated with type 2 diabetes status in human primary islets and in peripheral blood
Source: BMC Med Genomics. 2020 Apr 20;13:64. doi: 10.1186/s12920-020-0713-2 (PMC7171860; doi:10.1186/s12920-020-0713-2)
Supplement: Supplementary file 7 — Additional file 7. [file 12920_2020_713_MOESM7_ESM.docx]

**Supplementary table S5: Differential expression of the 5 most abundant islet circRNA with insulin secretory index (SI), donor HbA1c or T2D status.** We measured the expression of the 5 most abundantly-expressed circRNA and their linear counterparts in human pancreatic islet preparations with respect to **A.** Insulin secretory index (n = 50), **B.** Donor HbA1c (n = 18) or **C.** T2D status (n = 50 control islets and 20 islets from T2D donors). SD = Standard Deviation. 95% CI = 95% confidence intervals Transcripts demonstrating associations with glycaemic parameters that are significant following adjustment for multiple testing are given in bold underlined type. Those demonstrating nominal associations only are indicated in bold italic type.

**A**

| **CircRNA** | **β-coefficient** | **p-value** | **95% CI** | | |
| --- | --- | --- | --- | --- | --- |
| *CAMSAP1* | 0.051 | 0.444 | -0.184 | - | 0.082 |
| *CircCAMSAP1* | 0.060 | 0.101 | -0.133 | - | 0.012 |
| *CIRBP* | 0.007 | 0.894 | -0.112 | - | 0.098 |
| ***CircCIRBP**** | ***-0.153*** | ***0.029*** | ***-0.289*** | ***-*** | ***-0.017*** |
| ***RHOBTB3**** | ***0.083*** | ***0.044*** | ***0.003*** | ***-*** | ***0.164*** |
| *CircRHOBTB3* | -0.017 | 0.64 | -0.088 | - | 0.055 |
| RPH3AL | 0.026 | 0.626 | -0.133 | - | 0.081 |
| *CircRPH3AL* | 0.034 | 0.532 | -0.075 | - | 0.143 |
| *ZKSCAN1* | 0.085 | 0.272 | -0.069 | - | 0.239 |
| *CircZKSCAN1* | 0.028 | 0.357 | -0.033 | - | 0.090 |

**B**

| **CircRNA** | **β-coefficient** | **p-value** | **95% CI** | | |
| --- | --- | --- | --- | --- | --- |
| *CAMSAP1* | -0.297 | 0.368 | -1.005 | - | 0.412 |
| *CircCAMSAP1* | 0.128 | 0.582 | -0.379 | - | 0.635 |
| *CIRBP* | -0.423 | 0.325 | -1.342 | - | 0.496 |
| *CircCIRBP* | -0.024 | 0.977 | -2.084 | - | 2.035 |
| *RHOBTB3* | 0.237 | 0.429 | -0.410 | - | 0.884 |
| *CircRHOBTB3* | -0.228 | 0.420 | -0.837 | - | 0.382 |
| *RPH3AL* | 0.25 | 0.423 | -0.424 | - | 0.925 |
| *CircRPH3AL* | 0.067 | 0.873 | -0.858 | - | 0.992 |
| *ZKSCAN1* | -0.096 | 0.872 | -1.402 | - | 1.210 |
| *CircZKSCAN1* | 0.067 | 0.793 | -0.490 | - | 0.623 |

**C.**

| **CircRNA** | **Mean** | **SD** | **Mean** | **SD** | **p-value** |
| --- | --- | --- | --- | --- | --- |
|  |  |  |  |  |  |
| ***CAMSAP1*** | **0.049** | **0.550** | **0.658** | **0.424** | **<0.001** |
| ***CircCAMSAP1*** | **-0.010** | **0.302** | **1.341** | **0.405** | **<0.001** |
| ***CIRBP*** | **-0.007** | **0.454** | **-2.218** | **0.434** | **<0.001** |
| ***CircCIRBP*** | **0.070** | **0.530** | **-1.996** | **0.266** | **<0.001** |
| ***RHOBTB3*** | **0.000** | **0.428** | **-0.034** | **0.380** | **<0.001** |
| *CircRHOBTB3* | -0.009 | 0.425 | 0.823 | 0.494 | 0.419 |
| *RPH3AL* | 0.000 | 0.333 | 0.452 | 0.274 | 0.891 |
| ***CircRPH3AL*** | **-0.048** | **0.303** | **-0.001** | **0.377** | **<0.001** |
| ***ZKSCAN1*** | **-0.216** | **0.663** | **0.907** | **0.510** | **<0.001** |
| ***CircZKSCAN1**** | ***0.019*** | ***0.267*** | ***0.211*** | ***0.537*** | ***0.012*** |
|  |  |  |  |  |  |
