# Supplementary material for: Islet-expressed circular RNAs are associated with type 2 diabetes status in human primary islets and in peripheral blood
Source: BMC Med Genomics. 2020 Apr 20;13:64. doi: 10.1186/s12920-020-0713-2 (PMC7171860; doi:10.1186/s12920-020-0713-2)
Supplement: Supplementary file 8 — Additional file 8. [file 12920_2020_713_MOESM8_ESM.docx]

**Supplementary table S6: The expression of islet circRNAs according to diabetes status in the peripheral blood of individuals with IGT or overt T2D, compared with non-diabetic controls.** The expression of the *circCAMSAP1*, *circCIRBP* and *circZKSCAN* circular RNAs is given here in relation to **A**. fasting blood glucose (n = 130) and **B**. HbA1c (n = 80) in the peripheral blood of non-diabetic controls. **C.** The expression of the *circCAMSAP1*, *circCIRBP* and *circZKSCAN* circular RNAs is also given in relation to diabetes status in the peripheral blood of patients without disease (n =133), those with IGT (Impaired glucose tolerance; n = 46) and those with overt T2D (n = 106). SD = Standard deviation. Results showing statistical significance are indicated in bold italic type.

**A**

| **CircRNA** | **β-coefficient** | **95% CI** | | |  | | **p-value** |
| --- | --- | --- | --- | --- | --- | --- | --- |
| *CircCAMSAP1* | 0.069 | -0.085 | - | 0.222 | | 0.378 | |
| *CircCIRBP* | 0.039 | -0.276 | - | 0.353 | | 0.808 | |
| *CircZKSAN1* | 0.052 | -0.135 | - | 0.238 | | 0.585 | |

**B.**

| **CircRNA** | **β-coefficient** | **95% CI** | | |  | | **p-value** |
| --- | --- | --- | --- | --- | --- | --- | --- |
| *CircCAMSAP1* | -0.012 | -0.269 | - | 0.245 | | 0.928 | |
| *CircCIRBP* | 0.303 | -0.105 | - | 0.710 | | 0.143 | |
| *CircZKSAN1* | -0.257 | -0.558 | - | 0.044 | | 0.093 | |

**C.**

|  |  | **Control** | | **Case** | |
| --- | --- | --- | --- | --- | --- |
| **Transcript** | **p-value** | **Mean** | **SD** | **Mean** | **SD** |
| **Control vs T2D** | | | | | |
| ***CircCAMSAP1*** | ***0.029*** | ***-0.04*** | ***0.32*** | ***-0.10*** | ***0.32*** |
| *CircCIRBP* | 0.260 | -0.03 | 0.64 | -0.11 | 0.53 |
| *CircZKSCAN1* | 0.054 | 0.03 | 0.39 | 0.05 | 0.37 |
| **Control vs IGT** | | | | | |
| *CircCAMSAP1* | 0.606 | -0.04 | 0.32 | -0.05 | 0.29 |
| *CircCIRBP* | 0.913 | -0.03 | 0.64 | -0.12 | 0.68 |
| *CircZKSCAN1* | 0.987 | 0.03 | 0.39 | 0.06 | 0.34 |
| **IGT vs T2D** | | | | | |
| *CircCAMSAP1* | 0.495 | -0.05 | 0.29 | -0.10 | 0.32 |
| *CircCIRBP* | 0.281 | -0.12 | 0.68 | -0.11 | 0.53 |
| *CircZKSCAN1* | 0.089 | 0.06 | 0.34 | 0.05 | 0.37 |
